# Three-dimensional architecture of granulosa cell derived from oocyte cumulus complex, revealed by FIB-SEM

**DOI:** 10.1186/s13048-023-01298-9

**Published:** 2023-11-09

**Authors:** Chongyi Shu, Yiqi Yu, Xiaopan Chen, Jiansheng Guo, Yier Zhou, Dandan Wu, Tianyun Yang, Yuhang Fan, Qiongxiao Huang, Jing Shu

**Affiliations:** 1 Center for Reproductive Medicine, Department of Reproductive Endocrinology, Zhejiang Provincial People’s Hospital (Affiliated People’s Hospital, Hangzhou Medical College), Hangzhou, China; 2 Department of Genetic and Genomic Medicine, Zhejiang Provincial People’s Hospital, (Affiliated People’s Hospital, Hangzhou Medical College), Hangzhou, China; 3 School of Medicine, Zhejiang University, Hangzhou, 310058 China; 4 Department of Obstetrics, Zhejiang Hospital, Hangzhou, 310012 China

**Keywords:** Oocyte cumulus complex, Granulosa cells, Focused ion beam scanning electronmicroscopy, 3D reconstruction, Organelles, Intercellular connections

## Abstract

The oocyte cumulus complex is mainly composed of an oocyte, the perivitelline space, zona pellucida and numerous granulosa cells. The cumulus granulosa cells (cGCs) provide a particularly important microenvironment for oocyte development, regulating its growth, maturation and meiosis. In this study, we studied the internal structures and cell-to-cell connections of mouse cGCs using focused ion beam scanning electron microscopy (FIB-SEM). We reconstructed three-dimensional models to display characteristic connections between the oocyte and cGCs, and to illustrate various main organelles in cGCs together with their interaction relationship. A special form of cilium identified in granulosa cell was never reported in previous literature.

## Introduction

A high-quality oocyte is the start of success for prenatal and postnatal life. In mammals, follicles act as the basic units for the oocyte development [1]. Basic on developmental stages, follicles are categorized as primitive, primary, secondary, antral or mature. When a follicle develops to antral stage, the oocyte and its surrounding granulosa cells form a compact complex named the oocyte cumulus complex (OCC) protruding from the inside wall to the antrum of the follicle. The OCC, is composed of the oocyte, the perivitelline space, zona pellucida (ZP) and numerous cumulus granulosa cells (cGCs), from inside to outside. Just like the feeding cells, the cGCs regulate the development, maturation and meiosis of the oocyte through molecular communication, nutrient transport and energy provision [2–4]. They also coordinate the whole reproductive process in response to oocytogenic factors, follicle paracrine signals and endocrine hormones. In recent years, in vitro fertilization (IVF) has flourished and solved a lot of infertility problems. However, the live birth rate still hovers at 30 − 40%, far from satisfaction. A lot of failures are due to setbacks in oocyte retrieval, fertilization or embryonic development. Studies have shown that most of those failures were associated with the abnormal structure of oocytes or OCCs [5, 6]. Therefore, observation and analysis of OCC structure is the cornerstone of understanding the causes of corresponding types of infertility.

Ultrastructure of OCCs was revealed over half a century ago [7]. Most studies took close look into OCCs and their accessory organelles by transmission electron microscopy (EM) or scanning EM [7–9]. However, traditional EM techniques failed to reconstruct fine microstructural features in three-dimension (3D) view due to limitation of imaging methods in which plane figures were obtained by collecting signals from electron beams penetrating or reflexing to ultrathin, resin-embedded samples. In 1988, the first focused ion beam scanning electron microscope (FIB-SEM) was introduced and perfectly overcome the above limitation of traditional EM techniques. Not until the end of the twentieth century did scientists realize the value of FIB-SEM in biology. In 2013, Schertel et al. successfully reconstructed the mouse optic nerve and spores of Bacillus subtilis at freezing temperature using FIB-SEM, demonstrating the potential of FIB-SEM to image the ultrastructure of organisms in their natural state [10, 11]. By means of gallium ion beam sputtering trimming tissue accurately sputtering tissue with gallium ion beam, FIB-SEM collects continuous serial surface scanning images with z-axis high-resolution. After digital reconstruction, precise 3D structure images can be obtained [12–14]. The resolution level in FIB-SEM 3D images is high enough to visualize all organelles and macromolecular complexes, making it a powerful tool for biological research [15].

In this study, by using FIB-SEM, we investigated the OCCs and the organelles inside cGCs, and developed the 3D structure model for the first time. The 3D images revealed not only information about individual granulosa cell organelles, but also the spatial relationship between organelles and between cells, providing an approach to understanding the OCC metabolism mechanism. Furthermore, we discovered a special ciliary structure in granulosa cells, which was never described in previous literature. These updates in structural data may bring insights into OCC structure and morphogenesis.

## Materials and methods

### Experimental animals and animal handing

C57BL/6J mice were purchased from shanghai SLAC Animal Laboratory. The mice were housed in cages and maintained under a constant 12 h-bright and 12 h-dark cycle at 21 − 23 ºC with unlimited access to standard chow and water in a specific pathogen-free (SPF) vivarium at Zhejiang Provincial People Hospital. Four-week-old female mice were injected intraperitoneally with PMSG to promote follicular development. 16 − 18 h later, they were dissected to collect ovaries. This work was approved by the Institutional Review Board of Zhejiang Province People's Hospital.

### Intraovarian follicle isolation

The mouse ovarian tissue was cleaned with D-Hanks. After separating the surrounding connective tissue, the ovaries were cut into cubes at about 1mm^3^. The tissue cubes were transferred into EP tubes, mixed with collagenase I (2 mg/ml) and DNase (2 mg/ml) for digestion in a 37 °C, 5%-CO_2_ incubator for 60 min, and blown with a pipette for about 4 times every 15 min. After that, the suspension was filtered to obtain individual follicles. Multiple follicles with different diameters (< 40 μm, 40-100 μm, > 100 μm) were isolated. Based on morphology and diameter characteristics, we selected one representative sample from the follicles, which is a mature follicle.

### Samples and preparation for FIB-SEM

All samples were collected quickly and fixed for 24 h in 2.5% glutaraldehyde with 0.1 M phosphate buffer. They were washed in phosphate buffer (0.1 M) and treated with a solution containing equal volumes of 1% OsO_4_ and 1.5% potassium ferrocyanide for 1 h. The samples were then rinsed with double-distilled water (ddH_2_O) and treated with 2% aqueous OsO_4_ for 30 min at room temperature. After rinsing with ddH_2_O, the samples were immersed in 1% aqueous uranyl acetate overnight at 4 °C. Following that, they were dehydrated sequentially with a series of gradient acetone (50% − 70%-90% − 100%, each for 20 min). Then the samples were infiltrated with a mixed solution containing acetone and Epon 812 (1:1) and spun overnight. Lastly, the mixed solution was replaced with Epon 812 resin to embed the samples at 60 °C overnight.

### FIB-SEM data collection

Cured resin blocks were manually trimmed to expose the samples using a glass knife. The final block size was made as small as possible (about 1 mm^3^ in size) to minimise sample drift from continuous degassing of resin. To obtain the area of interest, we used a scanning electron microscope (SEM) (Thermo Fisher, Helios UC G3), which allowed synchronous tissue trimming and surface scanning imagery. The electron beam (SEM portion) helped to select and locate the site of interest, then the ion beam (FIB portion) was applied to prepare the block face for automated image stack acquisition. In this study, the data were collected from serial surface view mode, with slice thickness at 9 nm, at 30 keV and 0.79 nA. Each serial face was then imaged with an acceleration voltage of 2 kV and a current of 0.2 nA, in backscatter mode, with an in-column backscatter electron detector. We obtained images with 6144 × 4096 pixels, at a resolution of 9 nm per pixel. The resolution of FIB-SEM is nearly 1000 times higher than that of ordinary optical microscopes, making it possible to directly distinguish and classify cells upon the subcellular structures.

### Image processing and segmentation

The image stack was aligned and cropped, using Amira 6.5 (Thermo Fisher). The granulosa cell organelles were segmented and labeled manually. Surface-generation tools were used to compute the surfaces, then these surface files were simplified to 10% of the original value and saved as MRC Stack files (.mrc). All the resulting files were reassembled, colored and rendered into images in Chimera1.14.

## Results

### Oocyte Cumulus Complex Structure

**Fig. 1:**
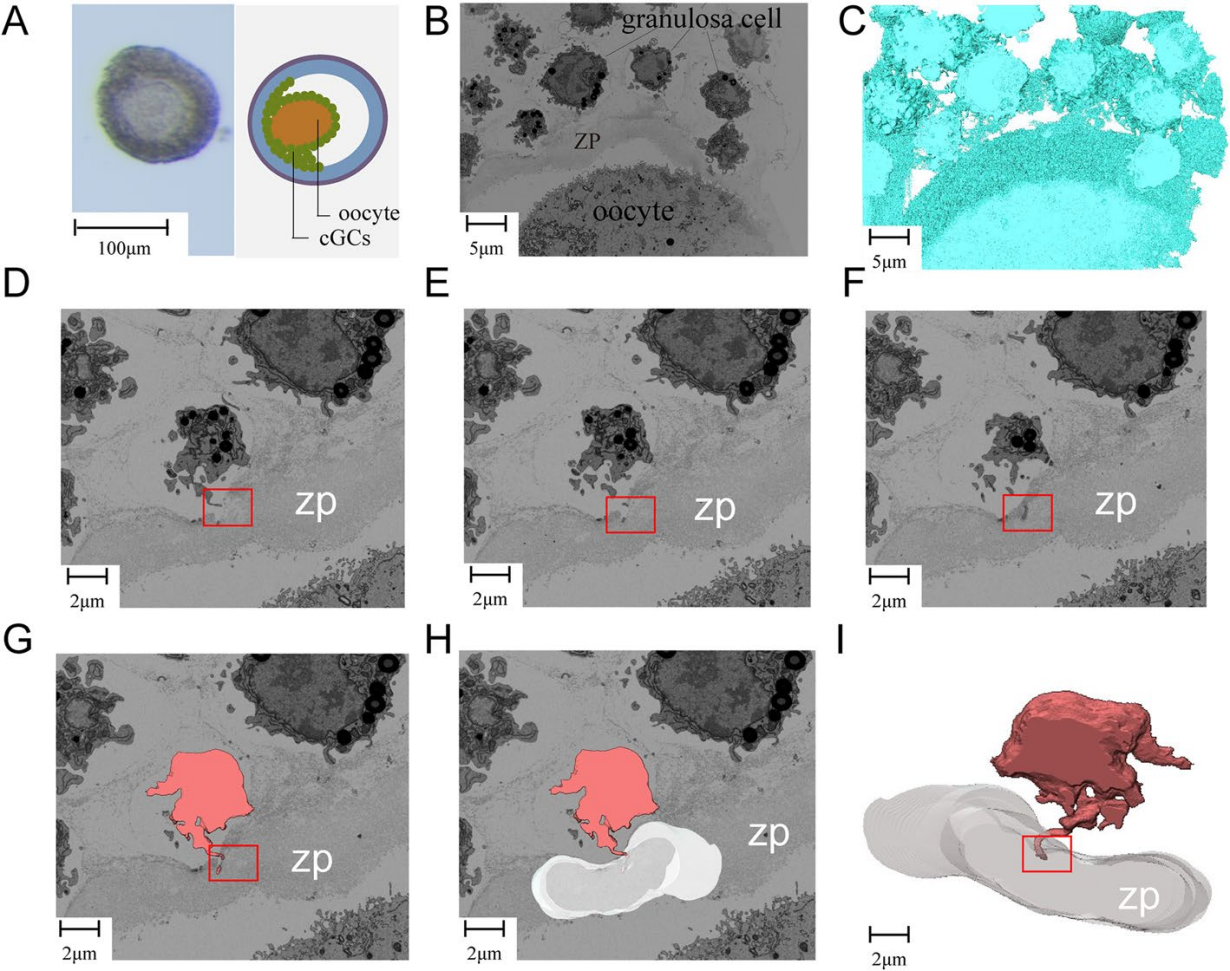
Partial structure of oocyte cumulus complex (OCC). **A** Light microscope image of a follicle with a diameter of about 135 μm (left). A schematic diagram (right) illustrates the follicle structure. **B** 2D SEM image of a part of OCC. **C** Reconstructed 3D model of the same part of the OCC. **D-F** Three consecutive SEM image sections focusing on the same granulosa cell with the transzonal projections (TZP) structure. **G-H** Reconstructed SEM image of Fig. 1d highlighting the granulosa cell, TZP and zona pellucida. **I** Reconstructed 3D model of a cGC with TZP. The red rectangular box represents the TZP structure

A typical follicle with a diameter of 135 μm was selected for analysis (Fig. 1A). The two-dimensional (2D) picture of the OCC is presented (Fig. 1B), focusing on the cGCs, zona pellucida, the perivitelline space and a part of the oocyte. The reconstructed 3D structure model gives a more straightforward illustration (Fig. 1C). Most granulosa cells in the OCC are round or elliptical with an average diameter of 9 μm. Both cGCs and the oocyte have continuous cytoplasmic membrane with great abundance in thin cytoplasmic projections, or so-called microvilli. These microvilli are often seen in touch with each other between cells (Fig. 1C). The granulosa cells adjacent to ZP possess characteristically wide and long microvilli penentrating the ZP, as denmonstrated clearly in our reconstructed 2D pictures (Fig. 1G − H) and 3D model (Fig. 1I).

### Intracellular structure of cumulus granulosa cells

**Fig. 2:**
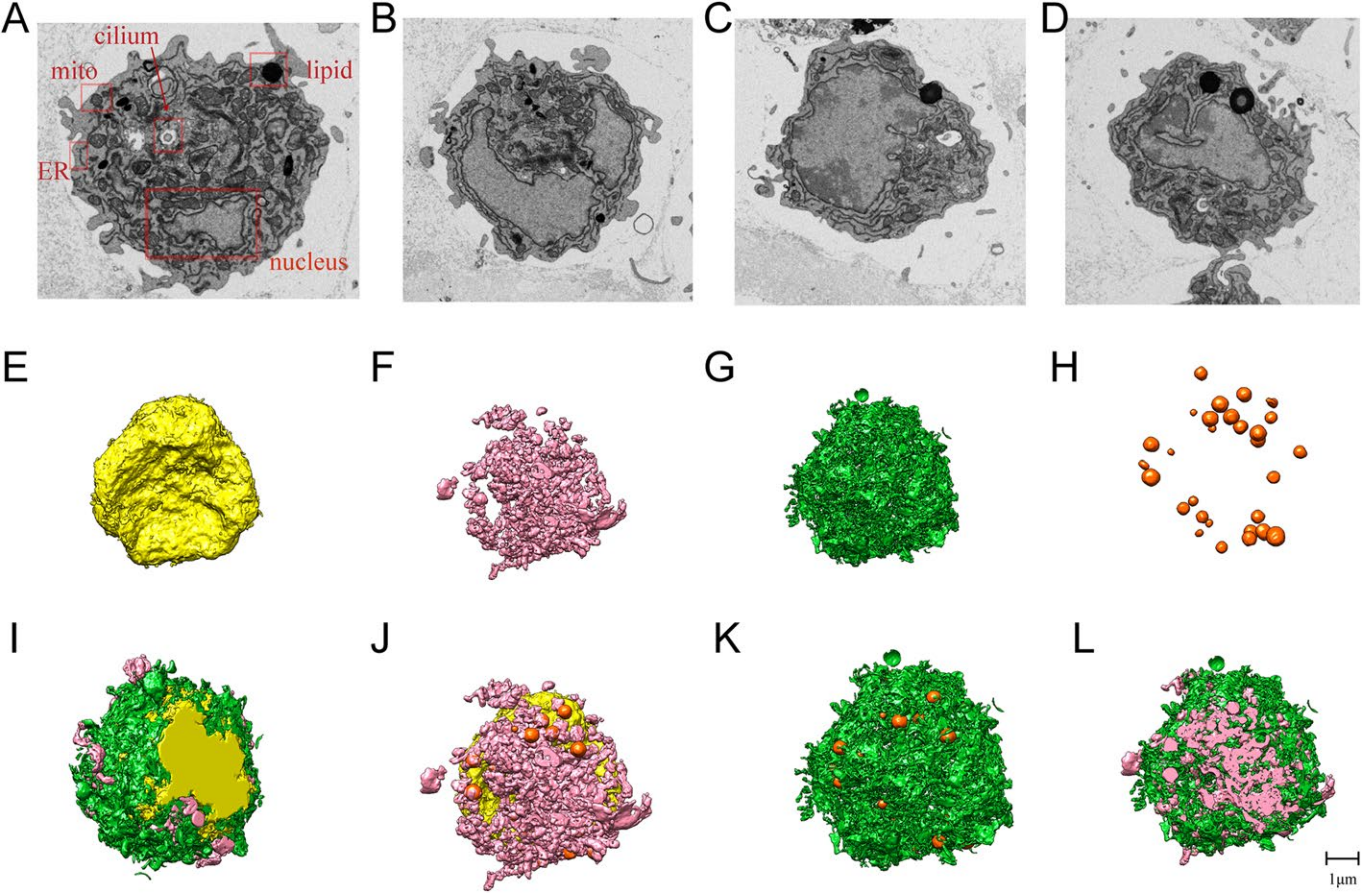
Models of individual organelles of cumulus granulosa cells (cGC). **A** SEM image of several characteristic organelles. **B**,**C**,**D** SEM images of three different cGCs showing large nuclei in cells. **E** 3D model of nuclear structure. **F** 3D model to show rod- and thread-shaped mitochondria. Most of them clustered together, and a few scattered (colored with pink). **G** 3D model to show endoplasmic reticulum (ER) with net-like structure and a sheet-like or tubular cavity (colored with green). **H** 3D model to show lipid droplets in different sizes (colored with orange). **I**,**J**,**K**,**L** 3D model to show spatial relationship between organelles in a cGC. Note the close contact between mitochondria and ER, warpped the nucleus with lipid droplets scatted peripheral

In this study, three typical granulosa cells adjacent to ZP had been selected for organelles observation (Fig. 2). The FIB-SEM section shows a large, round and deep-colored nucleus, separated from the cytoplasm by a continuous electron-dense nuclear membrane (Fig. 2A). The nucleus has a double-layered nuclear membrane, adsorbing more heavy metal and rebounding more electron density. Therefore the nucleus appeared more intense than other organelles. Abundant organelles, especially mitochondria, endoplasmic reticulum (ER) and lipid droplets (Fig. 2B − D), are seen in the cytoplasma. Mitochondria are mainly irregularly round or elongated with many tightly packed tubule vesicle cristae. ER is composed of tubular and vesicular membrane structure. Lipid droplets are spherical structures with high electron density, always located near the cell membrane.

Labelled by Amira 6.5 software, 3D images of these granulosa cell had been reconstructed. In 3D view, the nucleus is not fully round, and its surface undulating and wrinkled. A large depression is seen on one side (Fig. 2E). The cell is rich in mitochondria, which appear in tubular, linear or oval shape. Most of them are aggregated, only a few scattered (Fig. 2F). ER is the largest organelle, which is continuous membrane-bounded network structure without free fragments (Fig. 2G). There are abundant lipid droplets. Most of them are smooth and spherical, diameters ranging from 50 to 800 nm, and the rest appear dumbbell-shaped or irregular (Fig. 2H).

Putting all the 3D models of intracellular organelles above together and looking as a whole (Fig. 2I − L), we can see that ER forms multiple layers of concentric circles embracing the nucleus. Mitochondria also intersperse around nucleus and attach to ER closely, formed mitochondria smooth ER (M-SER) aggregates and mitochondrial vesicle (MV) complexes. The lipid droplets distribute near the periphery of mitochondria. The calculated volume of a single nucleus, total mitochondria and total ER is 1.4 × 10^11^nm^3^, 3.5 × 10^10^nm^3^ and 3.1 × 10^10^ nm^3^ respectively, about 36%, 9% and 8% of the whole cell. The total area of mitochondria and ER are 3.5 × 10^6^nm^2^ and 9.1 × 10^6^ nm^2^ respectively. The average total number of mitochondria is 288 per granulosa cell.

**Fig. 3:**
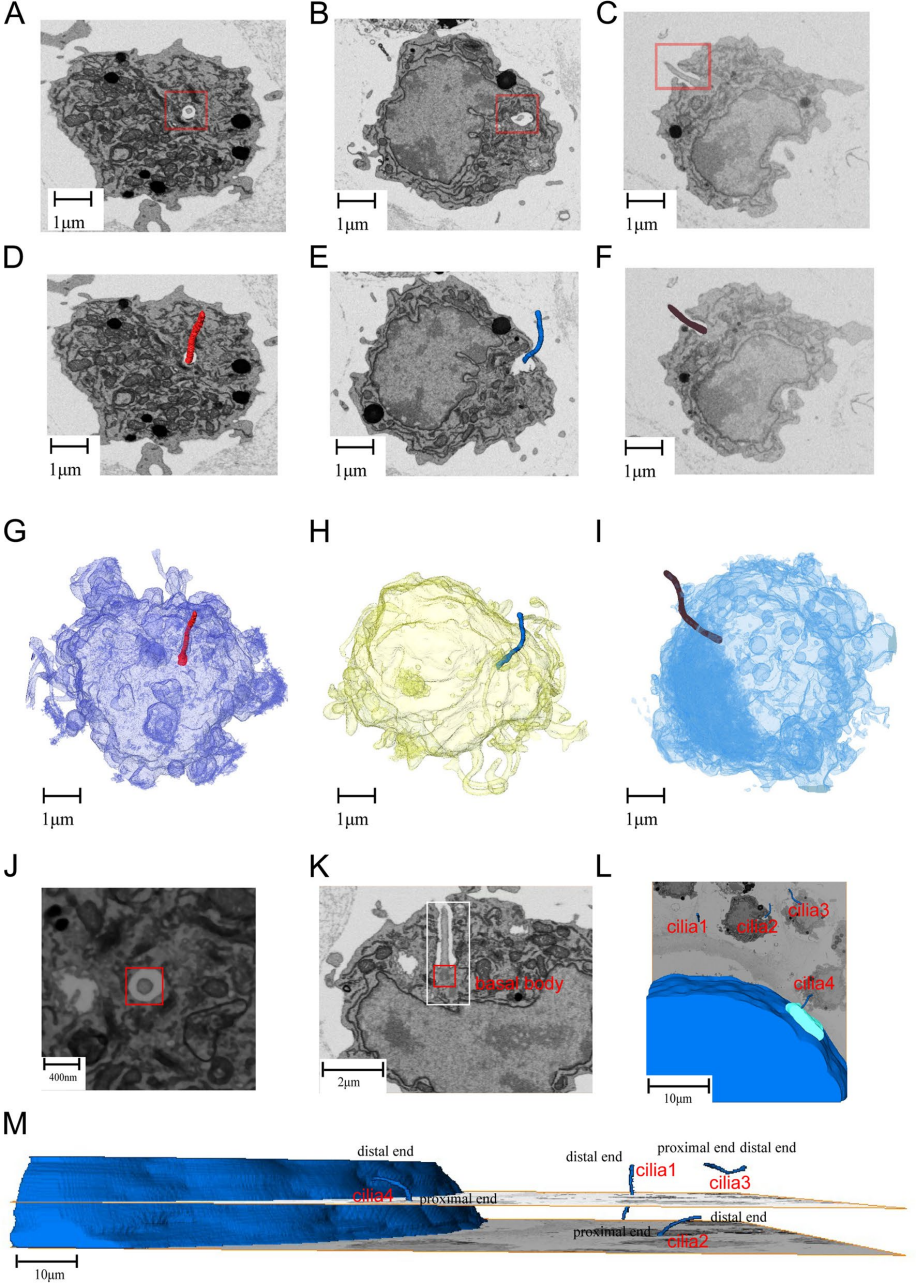
Structural model of the special ciliary structure. **A**,**B**,**C** SEM images of three cumulus granulosa cells (cGCs) with characteristic cilia at transverse sections (a,b) and longitudinal section (c). **D**,**E**,**F** Reconstructed ciliary structures in their corresponding cGCs. **G**,**H**,**I** 3D model to show spatial relationship of cilia in cGCs with color transparency. **J** Magnified image of the transverse section of the distal cilium showing the absence of 9 + 2 structure.** K** Magnified image of the longitudinal structure of the distal cilium showing basal body at its root (red box). **L** Reconstructed model to show the different orientation of cilia in cGCs around the oocyte. **M** Horizontal schematic displaying the spatial position and directions of the cilia of four cGCs

By FIB imaging, a special ciliary structure was discovered in granulosa cells for the first time. This structure was found in all the three cGCs we chose (Fig. 3A-C). It is an elongated straight spindly-shaped structure, extending from deep inside of cytoplasm and protruding out of the cell surface. The spatial localization of cilia in cells is well illustrated after further 3D reconstruction and transparentizing of the granulosa cells (Fig. 3D − I). The average root diameter, total area and volume of cilia are 250-300 nm, 4 × 10^6^nm^2^ and 1.5 × 10^8^nm^3^, respectively (Fig. 3G − I). After ffurther magnification of the images, we discovered that the 9 + 2 structure is absent at the projecting segment in transverse section (Fig. 3J). From longitudinal section, basal body is seen at the root of the cilium near daughter centrioles (Fig. 3K), docking the cilium to the cellular membrane through its distal appendages (Fig. 3K). We performed 3D reconstruction on cilia of a group of adjacent cGCs and revealed that the cilia were in different directions (Fig. 3L − M). One cilium was noted to be penetrating the zona pellucida.

### Spatial relationship between ER, mitochondria and lipid droplets

Most mitochondria are tubular, linear, or oval granular. They are vesicular bodies surrounded by a double-layered unit membrane. The inner membrane extends into the mitochondrial matrix and forms a lamellar or tubular inner fold called ridge. The endoplasmic reticulum is strip-shaped, block-shaped, or unilateral ring-shaped, which can reconstruct to form a network structure [16].

**Fig. 4:**
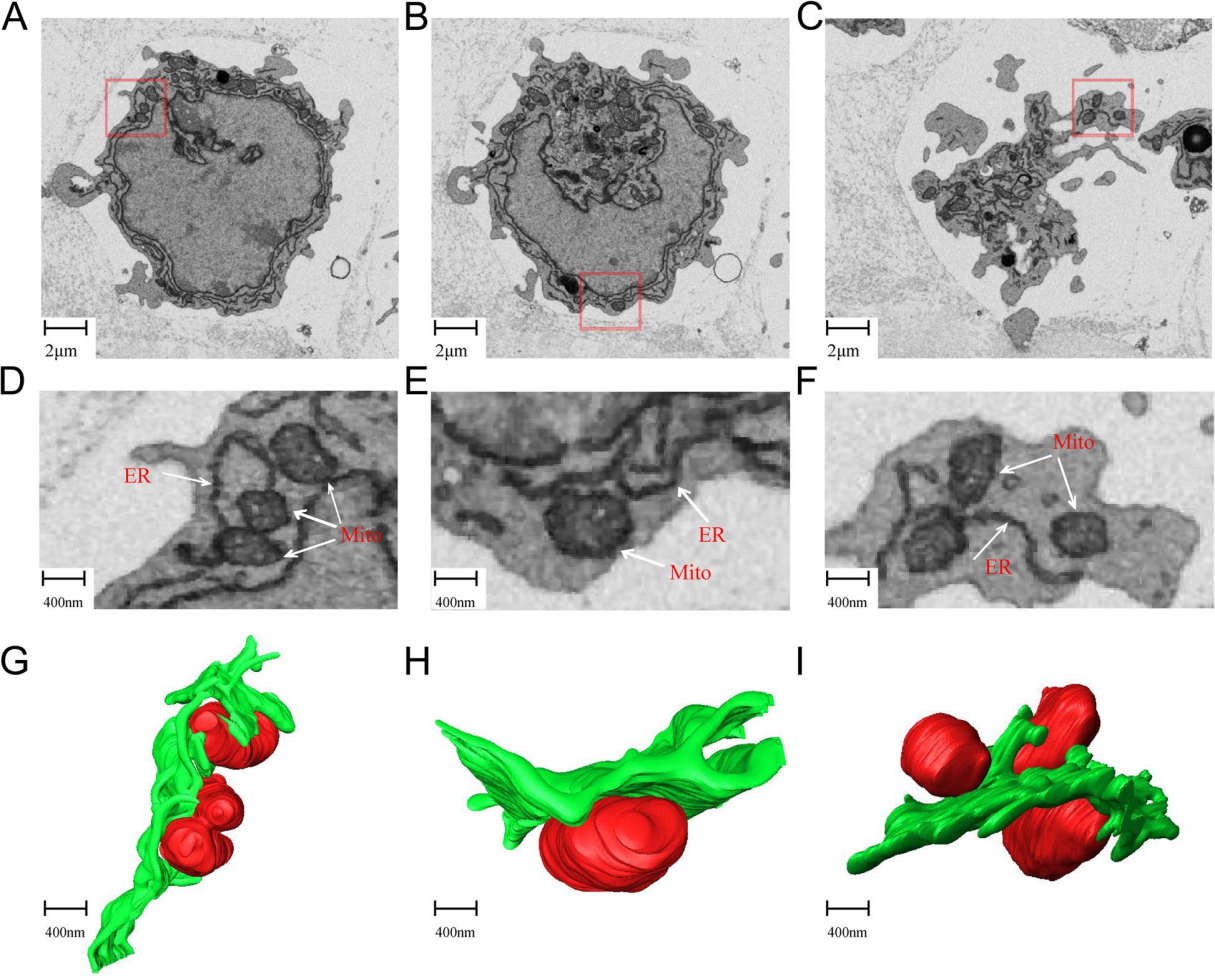
Reconstruction model to illustrate interaction between mitochondria and endoplasmic reticulum (ER) in cumulus granulosa cells (cGCs). **A**,**B**,**C** Three representative SEM images to show interaction of mitochondrial and endoplasmatic reticulum in two cGCs. **D**,**E**,**F** Zoom-in views of red boxes in a,b,c, respectively. **G**,**H**,**I** 3D reconstructed models of d,e,f, respectively. (Red: mitochondria; green: ER)

As showed in Fig. 2, mitochondria and ER are closely related within cGCs. In order to understand their interaction in detail, we selected images of three typical cGCs for further analysis. All the three cells present a lot of connections between mitochondria and ER (Fig. 4A − C). It can be seen clearly on the magnified image that several portions of mitochondrial outer membranes touch ER membrane (Fig. 4D − F), forming a mitochondrial-ER structure coupling. At these sites, the distance between the membranes of the two organelles is about 9-36 nm. This coupling is usually associated with functions such as calcium signal regulation, phospholipid synthesis and transport. In addition, a lot of mitochondria are almost "inlaid" in the depressions of the ER (Fig. 4G, H). Such spatial relationship may play a role in regulation of mitochondria function and replication. Furthermore, some tubular ER distorts mitochondria (Fig. 4I). One hypothesis is that ER constantly squeezes mitochondria, causing regional shrinkage, and leading to mitochondrial division and fusion.

Lipid droplets are composed of a neutral lipid core with a small amount of free cholesterol wrapped by a phospholipid monolayer. The endoplasmic reticulum membrane is surrounded by droplets, indicating that lipid droplets are closely related to the endoplasmic reticulum. In addition, there is evidence that eukaryotic biological lipid droplets originate from the endoplasmic reticulum [17]. Lipid droplets are also spatially close to mitochondria in the mature granulosa cells of mice. The mitochondria surround the lipid droplets and there is often direct contact. It was noted that mitochondria tend to make contact with large lipid droplets as compared to small lipid droplets. This gives a clue that the interaction between lipid droplets and mitochondria [18] is under some regulation.

### Interaction between cGCs

**Fig. 5:**
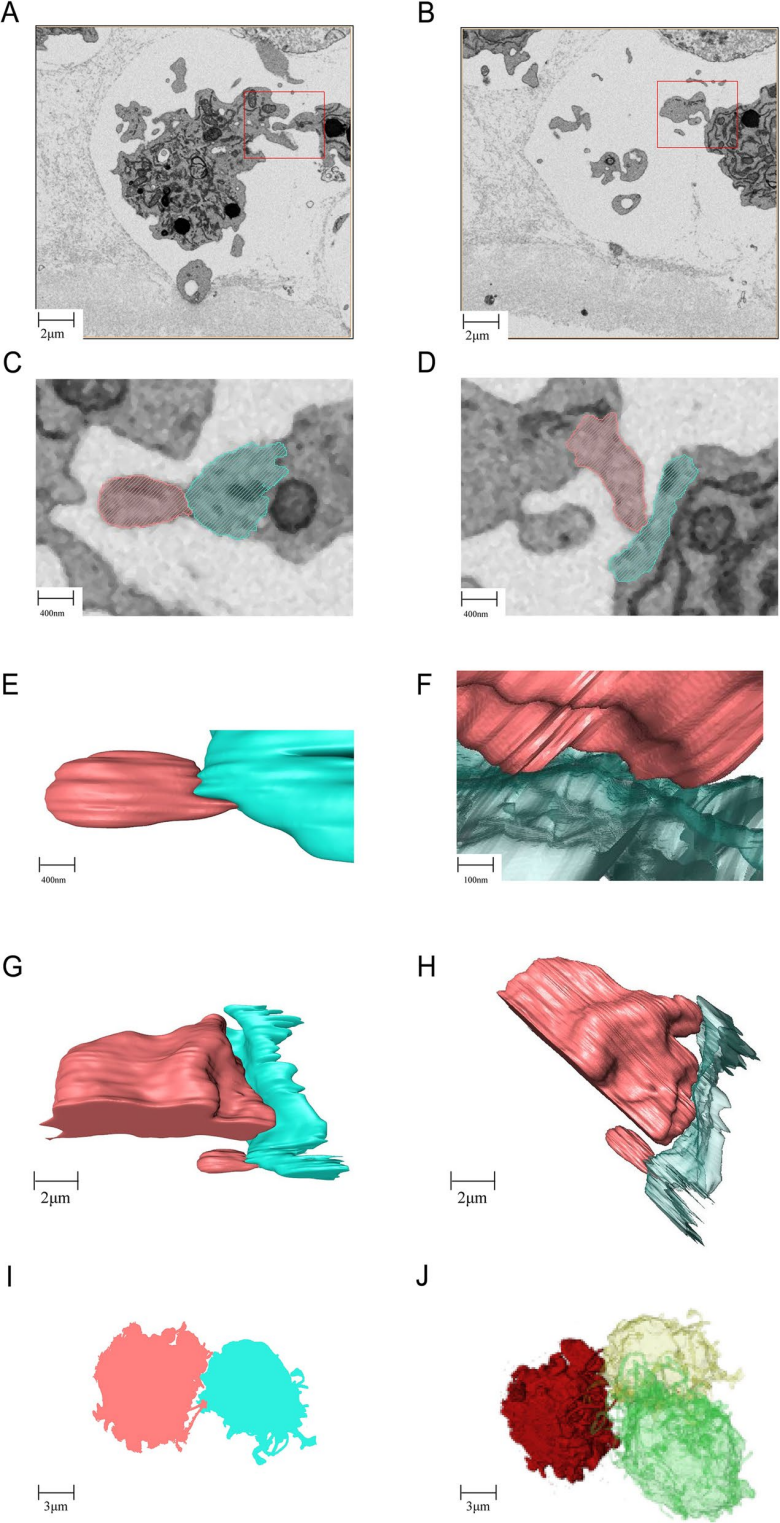
3D model to show the interaction between representative cumulus granulosa cells (cGCs). **A**,**B** SEM images of different contact sites between two cGCs. **C**,**D** Zoom-in views of a and b respectively. **E**,**F** Reconstructed images to show the membrane contact according to c and d respectively. **G**,**H** Reconstructed 3D model to show the parallel membranes with narrow gaps. **I** multiple gap junctions between two cGCs**. J** concentrated gap junctions seen at a small area where three cells met

The OCC contains a large number of cGCs. We cropped the original FIB-SEM images and picked out some adjacent cGCs of interest to study their intercellular interactions in detail (Fig. 5). Cell-to-cell linkage is seen between villi (Fig. 5A) or between a villium and smooth plasma membrane (Fig. 5B). This connection structure consists of adjacent cell membranes, local cytoplasma beneath the corresponding membrane and an intercellular part between the membranes (Fig. 5C, D). Revealed by the reconstructed 3D model, the adjacent cell membranes at the junction are highly parallel (Fig. 5E − F) and almost touching one another, with the intercellular gaps as narrow as 2-3 nm (Fig. 5G − H). This is evidence that gap junctions, rather than tight junctions, exist between granulosa cells. These junctions appear in groups at the interactive site (Fig. 5I), and may concentrate in a small area where two or three cells meet. (Fig. 5J).

## Discussion

Among various biological imaging methods, FIB-SEM exhibits obvious advantages in obtaining meticulous organelle information at a subcellular level with 3D view revealing entire cellular spatial distribution. Amira's precise algorithm not only produce 3D images efficiently, but also facilitates the measurement, calculation and comparation of number, area and volume on target research objectives. In this study, combining computer image processing with FIB‐SEM, a large amount of comprehensive 3D information on cell substructures is obtained. The OCC, a special complex comprised of an oocyte and granulosa cells are described in 3D space. This is an elementary unit for female reproductive outcome because it is where oocyte growth, maturation, meiosis and ovulation take place. FIB-SEM 3D imaging technology helps us to further understand the mechanism of functional realization in OCCs.

### Differences between cells and extracellular matrix

Cells and extracellular matrix can be clearly distinguished in FIB-SEM images. 1) Based on the continuity of the structure. The cell structure was continuous in all images of FIB-SEM without interruption from the first slice to the last. Non cellular substances, however, were non-continuous and appeared randomly. 2) Based on the difference in electron density. The electron density of cellular structures was high, while the electron density of non-cellular structures was low. Under electron microscopy, the contrast between cellular structures and non-cellular structures was significant. 3) Based on the different morphology The cellular structure normally exists as an integrated structure, in the form of sheets or blocks, while the non-cellular structures (impurities) were mostly sparsely distributed dots.

### Interaction between cells

As can be seen in our results, OCC is a multicellular complex rich in intercellular connections. These connections are an important basis for interaction and synergy between adjacent cells. In general, there are four main types of cell connections: a) tight junctions with blocking effect, which usually close the intercellular space at tissue surface; b) intermediate junctions with adhesion effect, which maintain cell shapes and transmit contractile force from cell to cell; c) desmosomes with anchoring effect, which fix and support cells. d) gap junctions with communication effect, which facilitates intercellular exchange of certain small molecules and ions. Among the four types, only gap junctions were observed between cGCs and between oocytes and cGCs. On our 3D images, the adjacent plasma membranes at the junctions are very close to each other and extremely parallel, the distance is as short as 3 nm. The density and abundance in the microvilli-like protrusions on the surface of cGCs and oocytes facilitate the formation of extensive gap junctions between cells.

Previously confirmed by X-ray diffraction technology, the gap junction is a hexameric structure of membrane protein molecules. Six monomers constitute a central tubule protruding outwards from the cell membrane, directly opposite to the central tubule of the adjacent cell in a spatial symmetry pattern. These central tubules may help facilitate exchanges of ions and small molecules between cells, such as amino acids, glucose, nucleotides, vitamins, hormones, growth factors, cAMPs, etc. Unfortunately, the hexamer could not be observed at the FIB-SEM resolution level.

Granulosa cells differentiate throughout follicular development from flat to columnar. They first form monolayers then stratified layers, and then synthesize and secrete mucopolysaccharides surrounding the oocyte to assist zona pellucida formation. Meanwhile, the innermost layer consistently remains close contact with the oocyte to allow two-way communication [18]. The abundance in microvilli in both cGCs and oocytes were greater than we expected. The characteristic microvilli on the granulosa cells adjacent to ZP are called transzonal projections (TZPs). TZPs can pass through the ZP as thick as 3–5 μm and reach oolemma, forming gap junctions connecting cGCs and the oocyte.

As the follicle develops, the plentiful cross-belt membrane protrusions provide extensive gap junctions for better nutrients and signal transmission to regulate oocyte maturation [19]. The existence of gap junctions in OCCs is essential for follicles to develop, mature and respond to endocrine signals as a whole. In addition, gap junction is a dynamic structure [20], whose opening and closing can be regulated by many factors. For example, the pores close upon a decrease in membrane potential or pH, or an increase in calcium ions (Ca^2+^) concentration, to protect cells from damage.

### Organelles in cGCs

CGCs are the driver of follicular growth as they are responsible for energy supply and signal transmission [21]. Although appeared sphericalunder light microscopy observation, they are in fact in irregular shape. With the high resolution of the electron microscope, a large number of tentacles and pseudopods with different lengths can be seen on the cell membranes of cGCs. Oocytes, on the other hand are generally spherical even under electron microscopy, because they are plump and possess abundant microvilli on the surface of the cell membrane. In this study, we confirmed that cGCs have abundant typical energy and steroid producing organelles, such as mitochondria, ER and lipid droplets. Interaction is observed between these organelles.

In response to the need of follicular growth, oxidative phosphorylation is intensified in cGCs to produce sufficient ATP [22], accompanied with high mitochondrial membrane potential [23] and high ROS levels [24, 25]. In our images, the mitochondria possess tightly-packed tubular vesicle cristae and distribute close to ER, which is evidence of highly active oxidative phosphorylation. However, pathological granulosa cells are characterized with mitochondrial crista dissolution, fracture, and vacuole formation. More seriously, pathological granulosa cells exhibit obvious features of apoptosis [26]. Besides, the 3D images also revealed a lot of M-SER and MV structures, which may be involved in material or membrane reservoirs, and work as one of the predictive factors for subsequent fertilization and early embryogenesis. In cGCs, the mitochondria tightly surround the lipid droplets. The mitochondrial-associated membrane (MAM) not only forms a special part of ER but also constitutes the lipid synthesis center. Membrane contact sites between lipid droplets and mitochondria play a role in mitochondrial fatty acid transport and beta oxidation [27].

ER is the intracellular reservoir of Ca^2+^ and contains most of the biosynthetic enzymes involved in the synthesis of lipids. By regulating Ca^2+^ signal transduction, ER controls lipid synthesis and mitochondrial biogenesis. The cGCs with vigorous ER provide more energy and materials to the oocyte. The abundance in functional ER is an indicator of good-quality cGCs.

The association between oocyte ultrastructural abnormalities and infertility was first reported by Afzeliusin in 1955 [28]. Since then, EM technology has made a great contribution to the diagnosis of infertility-related diseases, but the majority only focuses on abnormal structure of oocytes. As our understanding of the whole follicle structure deepens, the abnormal morphology of cGCs may also provide valuable diagnostic information in the future.

### Discovery of cilia in granulosa cells

In this study, we discovered for the first time a special ciliary structure in granulosa cells. Cilia are microtubule-based hair-like organelles with different functions at different stages of a cell. During cell division, it acts as a centriole; during functional phase, it acts as a sensor. Cilia protrude out of cell surface to help cell move and sense signals. It can thus be divided into two categories: motor cilia and non-motor cilia [29]. Motor cilia have a typical 9 + 2 microtubule structure. Take the tail of sperm as an example, its dysfunction is related to impaired male reproductive tract development and male infertility [30]. Non-motor cilia are also called sensory cilia, made up of microtubules. They may act as antennas, receiving and converting signals in intercellular communication [31], which is essential in the development of tissues and organs.

Although cilia are reported to be ubiquitous in cells, their existence and function in granulosa cells are still controversial [32]. Our study showed that each of the analyzed cGCs had a cilium lacking a central microtubule. The cilia protrude out of the cell surface in random directions. We hypothesize that they may help the cGCs to perceive the external micro-environment and make adjustment according physiological activity.

Studies have shown that cilia have abundant Ca^2+^ permeable membrane channels, activation of which can cause Ca^2+^ influx. Is calcium channel the main sensing channel in granulosa cell cilia? What signals do the cilia sense? What kind of cell activities can they regulate? What is the role of cilia in luteinization of granulosa cells? What is the association between the cilia and infertility? All of these questions deserve further study.

## Conclusion

By conventional transmission and scanning EM, it is difficult to study the whole OCCs with regards to its morphology, intercellular relationship and intracellular organelles all at the same time because the OCCs are irregularly-shaped and information is limited on one single cross-section. FIB-SEM is a powerful technique to create fine 3D images, making it possible to collect global and local structural information of the OCCs. In this study, the general structural features of the OCCs, spatial relationship and connections between component cells, as well as the organelles of cGCs are displayed by FIB-SEM 3D reconstruction model. These images provide novel information in addition to previous conventional EM studies and help us further understand follicular physiology.

## References

[1] McGee EA, Hsueh AJ (2000). Initial and cyclic recruitment of ovarian follicles. Endocr Rev.

[2] Chronowska E (2014). High-throughput analysis of ovarian granulosa cell transcriptome. Biomed Res Int.

[3] Gilchrist RB, Lane M, Thompson JG (2008). Oocyte-secreted factors: regulators of cumulus cell function and oocyte quality. Hum Reprod Update.

[4] Dumesic DA (2015). Oocyte environment: follicular fluid and cumulus cells are critical for oocyte health. Fertil Steril.

[5] Stetson I, Avilés M, Moros C, et al. Four glycoproteins are expressed in the cat zona pellucida. Theriogenology. 2014;83(7):1162–73. 10.1016/j.theriogenology.2014.12.019 25623231

[6] Sousa M (2015). Embryological, clinical and ultrastructural study of human oocytes presenting indented zona pellucida. Zygote.

[7] Segovia Y (2017). Ultrastructural characteristics of human oocytes vitrified before and after in vitro maturation. J Reprod Dev.

[8] Sathananthan AH (2013). Ultrastructure of human gametes, fertilization and embryos in assisted reproduction: a personal survey. Micron.

[9] Hafez ESE, Kenemans P (1983). Atlas-of-Human-Reproduction-By-Scanning-Electron-Microscopy. Book.

[10] Schertel A (2013). Cryo FIB-SEM: volume imaging of cellular ultrastructure in native frozen specimens. J Struct Biol.

[11] Briggman KL, Denk W (2006). Towards neural circuit reconstruction with volume electron microscopy techniques. Curr Opin Neurobiol.

[12] Schiel JA (2011). Endocytic membrane fusion and buckling-induced microtubule severing mediate cell abscission. J Cell Sci.

[13] Narayan K, Subramaniam S (2015). Focused ion beams in biology. Nat Methods.

[14] Trebichalska Z (2021). High-Resolution 3D Reconstruction of Human Oocytes Using Focused Ion Beam Scanning Electron Microscopy. Front Cell Dev Biol.

[15] Hayworth KJ (2015). Ultrastructurally smooth thick partitioning and volume stitching for large-scale connectomics. Nat Methods.

[16] Heinrich L (2021). Whole-cell organelle segmentation in volume electron microscopy. Nature.

[17] Daniele T, Schiaffino MV. Lipid transfer and metabolism across the endolysosomal–mitochondrial boundary. BBA. 2016;1861(8 Pt B):880–94. 10.1016/j.bbalip.2016.02.001 26852832

[18] Fu XW (2009). Positive effects of Taxol pretreatment on morphology, distribution and ultrastructure of mitochondria and lipid droplets in vitrification of in vitro matured porcine oocytes. Anim Reprod Sci.

[19] Mizumachi S (2018). Macromolecular crowded conditions strengthen contacts between mouse oocytes and companion granulosa cells during in vitro growth. J Reprod Dev.

[20] Zheng X (2021). LH upregulates connexin 43 expression in granulosa cells by activating the Wnt/beta-catenin signalling pathway. Reprod Fertil Dev.

[21] Matsuda F (2012). Follicular growth and atresia in mammalian ovaries: regulation by survival and death of granulosa cells. J Reprod Dev.

[22] Kansaku K (2017). Differential effects of mitochondrial inhibitors on porcine granulosa cells and oocytes. Theriogenology.

[23] Song ZQ (2017). DMBA acts on cumulus cells to desynchronize nuclear and cytoplasmic maturation of pig oocytes. Sci Rep.

[24] Jancar N (2007). Effect of apoptosis and reactive oxygen species production in human granulosa cells on oocyte fertilization and blastocyst development. J Assist Reprod Genet.

[25] Virant-Klun I (2018). Human oocyte maturation in vitro is improved by co-culture with cumulus cells from mature oocytes. Reprod Biomed Online.

[26] Zhao WP (2019). Mitochondrial respiratory chain complex abnormal expressions and fusion disorder are involved in fluoride-induced mitochondrial dysfunction in ovarian granulosa cells. Chemosphere.

[27] Strauss JA, Shaw CS, Bradley H, et al. Immunofluorescence microscopy of SNAP23 in human skeletal muscle reveals colocalization with plasma membrane, lipid droplets, and mitochondria. Physiol Rep. 2016;4(1):1–11. 10.14814/phy2.12662 PMC4760398 26733245

[28] Afzelius BA (1955). The ultrastructure of the nuclear membrane of the sea urchin oocyte as studied with the electron microscope. Exp Cell Res.

[29] Choksi SP (2014). Switching on cilia: transcriptional networks regulating ciliogenesis. Development.

[30] Girardet L, Augière C, Asselin MP, Belleannée C. Primary cilia: biosensors of the male reproductive tract. Andrology. 2019;7(5):588–602. 10.1111/andr.12650 31131532

[31] Brucker L, Kretschmer V, May-Simera HL (2020). The entangled relationship between cilia and actin. Int J Biochem Cell Biol.

[32] Kaur S, McGlashan SR, Ward ML (2018). Evidence of primary cilia in the developing rat heart. Cilia.

